# MDA5 with Complete CARD2 Region Inhibits the Early Replication of H9N2 AIV and Enhances the Immune Response during Vaccination

**DOI:** 10.3390/vaccines11101542

**Published:** 2023-09-28

**Authors:** Tongtong Li, Yiqin Cai, Chenfei Li, Jingwen Huang, Jiajing Chen, Ze Zhang, Ruibing Cao, Bin Zhou, Xiuli Feng

**Affiliations:** 1 Key Laboratory of Animal Microbiology of China’s Ministry of Agriculture, College of Veterinary Medicine, Nanjing Agricultural University, Nanjing 210095, China; 2018107083@njau.edu.cn (T.L.); 2021107050@stu.njau.edu.cn (Y.C.); 2019807121@njau.edu.cn (C.L.); 2020807124@stu.njau.edu.cn (J.H.); 2018107084@njau.edu.cn (J.C.); 2018107042@njau.edu.cn (Z.Z.); crb@njau.edu.cn (R.C.); zhoubin@njau.edu.cn (B.Z.); 2 MOE Joint International Research Laboratory of Animal Health and Food Safety, College of Veterinary Medicine, Nanjing Agricultural University, Nanjing 210095, China

**Keywords:** MDA5, H9N2 AIV, recombinant gene expression, antiviral, immune enhancement

## Abstract

Chicken melanoma differentiation-associated gene 5 (MDA5) is a member of the RLRs family that recognizes the viral RNAs invading cells and activates downstream interferon regulatory pathways, thereby inhibiting viral replication. The caspase activation and recruitment domain (CARD) is the most important region in MDA5 protein. However, the antiviral and immune enhancement of MDA5 with the CARD region remains unclear. In this study, two truncated MDA5 genes with different CARD regions, namely MDA5-1 with CARD1 plus partial CARD2 domain and MDA5-2 with CARD1 plus complete CARD2 domain, were cloned via reverse transcription PCR and ligated into plasmid Flag-N vector to be Flag-MDA5-1 and Flag-MDA5-2 plasmids. DF-1 cells were transfected with two plasmids for 24 h and then inoculated with H9N2 virus (0.1 MOI) for 6 h to detect the levels of IFN-β, PKR, MAVS, and viral HA, NA, and NS proteins expression. The results showed that MDA5-1 and MDA5-2 increased the expression of IFN-β and PKR, activated the downstream molecule MAVS production, and inhibited the expression of HA, NA, and NS proteins. The knockdown of MDA5 genes confirmed that MDA5-2 had a stronger antiviral effect than that of MDA5-1. Furthermore, the recombinant proteins MDA5-1 and MDA5-2 were combined with H9N2 inactivated vaccine to immunize SPF chickens subcutaneously injected in the neck three times. The immune response of the immunized chicken was investigated. It was observed that the antibody titers and expressions of immune-related molecules from the chicken immunized with MDA5-1 and MDA5-2 group were increased, in which the inducing function of MDA5-2 groups was the highest among all immunization groups. These results suggested that the truncated MDA5 recombinant proteins with complete CARD2 region could play vital roles in antiviral and immune enhancement. This study provides important material for the further study of the immunoregulatory function and clinical applications of MDA5 protein.

## 1. Introduction

The immune system serves as the first line of defense in recognizing and eliminating invading pathogens, utilizing pattern recognition receptors (PRRs) to identify these pathogens [1]. MDA5 is a member of the RLRs family and recognizes non-self RNA. There are three main regions in MDA5, namely two CARDs and one CTD domain [2]. CARD is a module containing 90–100 amino acids, which binds to the CARD of MAVS situated on the mitochondrial membrane [3]. The CARD–CARD interaction activates intracellular NF-κB and IRF3, thereby activating IFN-I pathway and pro-inflammatory cytokine secretion, which establishes a systematic antiviral system [3,4]. The RLRs themselves, encoded by interferon-stimulated genes (ISGs), constitute an anti-viral positive feedback pathway. IFN-I further orchestrates the cellular immune response to viral infection [5]. In addition to inducing type I interferons, RLRs and MAVS activate apoptosis, which removes the infected cells [6]. Thus, RIG-I and MDA5 are essential for antiviral immunity.

Mammalian RIG-I and MDA5 share a common signaling pathway involving the interaction with downstream MAVS; however, they identify different types of RNA. RIG-I mainly recognizes short double-stranded RNAs (<1 kbp) and single-stranded RNAs with uncapped 5′-triphosphate (5′-PPP), such as orthomyxoviruses (influenza A and B viruses), paramyxoviruses viruses (Sendai, Newcastle, and Measles viruses), flaviviruses (Hepatitis C and Japanese Encephalitis viruses), and fibro-viruses (Ebola virus). In contrast, MDA5 recognizes long dsRNAs (>2 kbp) and more complex structures, such as small RNA viruses (encephalomyocarditis virus) and certain negative-stranded RNA viruses (AIV). Dengue virus and West Nile virus, members of the Flaviviridae family, are recognized by two receptors. Differences in the ligand recognition of RNAs contribute to the identification of different pathogens [7,8].

It is reported that the RIG-I gene is not identified in chickens [9]. The absence of RIG-I receptors in chickens may lead to insufficient resistance to AIV infection, making chickens more susceptible to AIV infection than ducks with the RIG-I gene [9,10]. In mammals, RIG-I is the major cytoplasmic PRR required for the detection of AIV, and in chickens, MDA5 is the major cytoplasmic PRR required for the detection of AIV in chicken cells. ChMDA5 recognizes influenza A virus RNA, which induces the production of IFN-I, and chicken LGP2, MAVS, and IRF3 are also involved in this pathway. Therefore, the role of MDA5 in combating AIV has received extensive attention.

It was found that the expression of chicken IFN-β decreased when MDA5-deficient DF-1 cells were stimulated with different lengths of poly(I:C) [11]. These studies suggest that the partial compensation for the function of RIG-I of chicken MDA5 differs from that of murine MDA5.

Due to the poor immunogenicity of some inactive vaccine antigens, including subunit vaccines, the immune enhancement components are required to assist effector T cells and antibodies in producing adequate immune protection [12]. Most adjuvants enhance T- and B-cell responses through pathways involved in the innate immune system, rather than acting directly on the lymphocytes themselves [13,14]. The most commonly used adjuvants are immunostimulatory agents or carriers, with most immunostimulatory adjuvants being ligands for PRRs [15,16,17,18]. The RLR signaling pathway can enhance the adjuvant activity of Poly(I:C) [19]. The N-terminal CARD sequence derived from MAVS elicits a type I IFN response and possesses adjuvant properties [20]. In a mouse model, MAVS overexpression was shown to enhance DNA-induced cellular immune responses and provide resistance to H5N1 AIV infection [21]. Mammalian RIG-I and MDA5 share a common signaling pathway involving interaction with downstream MAVS [22].

To investigate the correlation between the CARD2 region in MDA5 protein and immune function on H9N2 subtype AIV, in this study, two truncated fragments of MDA5, MDA5-1 (CARD1+ partial CARD2) and MDA5-2 (CARD1+ and complete CARD2) were cloned from DF-1 cells. The antiviral functions of two truncated MDA5-1 and MDA5-2 proteins on H9N2 AIV were detected through eukaryotic overexpression, virus replication, and siRNA technologies. Furthermore, the immune-inducing roles of these truncated MDA5 proteins in chicken vaccinations were investigated through antibody production and cellular-related immune molecular expressions. This study will have certain referential significance for exploring the functional mechanism of the CARD2 region of MDA5 proteins.

## 2. Materials and Methods

### 2.1. Cells and Strains

DF-1 cells were provided by the Jiangsu Academy of Agricultural Sciences, which were cultured in DMEM medium (319-005-CL, Wisent Biotechnology, Nanjing, China) containing 10% EXC fetal bovine serum (FSP500, EXCEL bio, Taicang, China) at 37 °C with 5% CO_2_. The AIV strain (A/chicken/Shandong/LY1/2017(H9N2)) was preserved in our laboratory [23].

### 2.2. Construction of Expression Vector Containing MDA5 Target Gene

**Figure 1:**
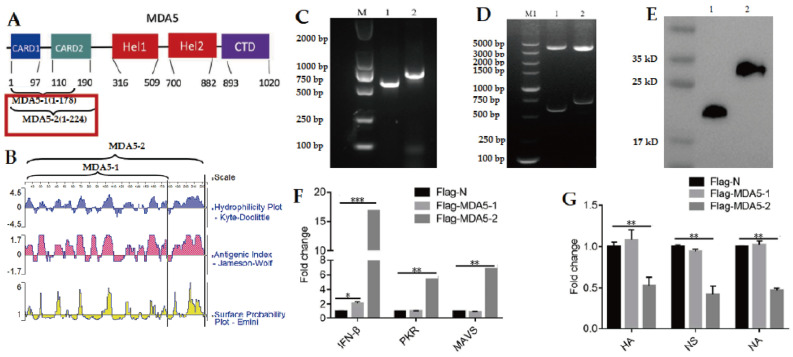
The transfection of MDA5 with CARD2 eukaryotic plasmid has an inhibitory effect on H9N2 virus replication. (**A**) The structure of MDA5 protein and design for truncated MDA5-1 and MDA5-2. (**B**) The protein characteristic analysis of MDA5-1 and MDA5-2. (**C**) The cloning of MDA5-1 and MDA5-2 genes. (**D**) The construction of the eukaryotic plasmids Flag-MDA5-1 and Flag-MDA5-2. (**E**) The eukaryotic expression of MDA5-1 and MDA5-2 proteins. (**F**) The transcript levels of IFN-β, PKR, and MAVS after transfection of MDA5-1 and MDA5-2 plasmids. (**G**) The transcription levels of HA, NS, and NA protein genes of H9N2 virus after MDA5-1 and MDA5-2 plasmid transfection. In (**C**–**E**), 1: MDA5-1 and 2: MDA5-2. * *p* < 0.05; ** *p* < 0.01; *** *p* < 0.001.

According to the reported CARD1 and CARD2 structures of MDA5 (Figure 1A) [2], two truncated MDA5 proteins were selected, which were named as MDA5-1 (from 1 to 178), with one CARD1 and a partial CARD2 region, and MDA5-2 (from 1 to 224), with CARD1 and complete CARD2 regions. The protein characteristics of MDA5-1 and MDA5-2 with the hydrophilicity, antigenic index, and surface probability were analyzed, as shown in Figure 1B.

The primers were designed according to the sequence of chicken-derived MDA5 genes (NM_001193638.1) in GenBank, as shown in Table 1. The total RNAs extracted from DF-1 cells were used as a template for PCR amplification. After reverse transcription, MDA5-1 and MDA5-2 genes were cloned via the following procedure: pre-denaturation at 94 °C for 5 min, denaturation at 94 °C for 50 s, annealing at 56 °C for 30 s, and extension at 72 °C for 30 s. After 35 cycles, the samples were extended at 72 °C for 5 min and detected in 1.5% agarose gel electrophoresis. Following double enzyme digestion with BamH I and Sal I, the gene fragments of MDA5-1 and MDA5-2 were ligated into the eukaryotic vector Flag-N-vector with T4 ligase at 16 °C overnight. The positive recombinant plasmids Flag-MDA5-1 and Flag-MDA5-2 were identified via double enzyme digestion and sequencing and then transfected into DF-1 cells. Whole-cell protein samples were collected after 24 h to detect the protein expression of MDA5-1 and MDA5-2 through Western blotting with anti-Flag antibody.

### 2.3. Cell Infection

When DF-1 cells were approximately 90% full in a 24-well plate, and 0.5 μg of Flag-MDA5-1 and Flag-MDA5-2 plasmids were transfected into DF-1 cells using Lipofectamine^®^ 3000 (L3000015, Thermo Fisher Scientific, Waltham, MA, USA) transfection reagent. At 24 h post-transfection, total cellular RNA was collected with Trizol to detect the expression of β-IFN, MAVS, and PKR genes. Additionally, DF-1 cells transfected with the plasmids for 24 h were inoculated with H9N2 virus (0.1 MOI). At 6 h post-transfection, the cellular RNA was extracted to determine the expression of viral HA, NA, and NS genes, of which the primer sequences are shown in Table 1.

### 2.4. Western Blotting

The protein samples were collected from the experimental groups and separated via SDS-PAGE. Target protein samples in polyacrylamide gel were electrically transferred onto the polyvinylidene fluoride (PVDF) membrane and blocked with 5% skim milk in PBST buffer (containing Tween 20) at 37 °C for 2 h. Subsequently, the membranes with the target samples were incubated with the corresponding primary antibody overnight and then incubated with HRP-goat anti-rabbit or HRP-goat anti-mouse secondary antibodies (BOSTER Biological CHNOLOGY, Pleasanton, CA, USA) at a dilution ratio of 1:10,000 in 5% skim milk for 1 h. Finally, the membranes were analyzed using ECL luminescent solution (170-5061, BIO-RAD, Hercules, CA, USA).

### 2.5. Fluorescence Quantitative PCR (qPCR)

The total RNAs were extracted from DF-1 cells treated with AIV, Flag-MDA5-1, and Flag-MDA5-2 and reverse-transcribed to be used as templates. β-actin was used as an internal reference, and fluorescence quantitative experiments were carried out together with the target genes, including β-IFN, MAVS, PKR, HA, and NS genes. The primer sequences are shown in Table 1. The data were calculated and processed using the following formula: Folg Change = 2^−ΔΔCt^, ΔΔCt = [Ct (test group target gene) − Ct (test group internal reference gene)] − [Ct (control target gene) − Ct (control internal reference gene)].

### 2.6. RNA Interference Experiments

For RNA interference experiments, two pairs of interfering primers were designed against the MDA5 genes, as shown in Table 1. About 80% filled DF-1 cells were transiently transfected with 100 μmol/L MDA5-siRNA1/2 and CTR-siRNA using the Lipofectamine^®^ 3000 (Thermo Fisher Scientific, Waltham, MA, USA) transfection reagent. At 36 h post-transfection, the levels of MDA5, β-IFN, and viral M gene were detected via RT-qPCR (TaKaRa, Beijing, China).

### 2.7. Prokaryotic Expression and Purification of MDA5-1 and MDA5-2 Proteins

MDA5-1 and MDA5-2 genes were cloned with the targeted primers to construct the prokaryotic expression plasmids pET28a-MDA5-1 and pET28a-MDA5-2. The primers are listed in Table 1. The constructed prokaryotic expression vectors were transferred to *BL*21(*DE*3) and induced with 1 mM IPTG overnight at 16 °C. After protein expression detection via SDS-PAGE and Western blotting, clean nickel packing was added to the protein supernatant and incubated via shaking at 4 °C for 4 h. The incubated supernatant was passed through the chromatography column, the heterogeneous proteins were eluted by washing the buffer with a low concentration of imidazole (50 mM Tris, 0.15 mM NaCl, and pH 8.0), and the target proteins were eluted by washing buffer with high imidazole concentration (200 mM and 400 mM). The concentration of the collected target protein was measured via the BCA (E112-01/02, Vazyme, Nanjing, China) method.

### 2.8. Preparation of Experimental Vaccine

The H9N2 subtype AIV was collected at 72 h after inoculation in 9-day-old SPF chicken embryos and inactivated with formaldehyde at 1:1000 of the virus solution volume at 37 °C for 24 h. After mixing with the purified MDA5-1 and MDA5-2 proteins, the viral antigen aqueous phase and oil adjuvant were thoroughly mixed at a ratio of 1:1 to prepare an oil emulsion inactivated experimental vaccine.

### 2.9. Chicken Immunization

Forty 1-day-old Hy-Line variety brown chickens were randomly divided into 5 groups as follows: AIV plus MDA5-1 (10 μg) and oil adjuvant, AIV plus MDA5-1 (20 μg) and oil adjuvant, AIV plus MDA5-2 (10 μg) and oil adjuvant, AIV plus MDA5-2 (20 μg) and oil adjuvant, and AIV plus BSA (10 μg) and oil adjuvant, in which the AIV plus BSA group was used as the antigen control. Chickens were subcutaneously injected in the neck with the experimental vaccine three times at two-week intervals.

### 2.10. Hemagglutination Inhibition Test

Serum samples were collected from all of the immunized chickens on the 14th day after the first and second immunization. The samples were serially diluted twofold in a 96-well V-bottom plate, and 25 μL of 4HA per well was added into plates. After incubation for 30 min at room temperature, 50 μL of 1% chicken erythrocytes were added into each well for 30 min. Then, the antibody levels were calculated, in which HI titers were expressed as the reciprocal of the highest dilution, expressed as log2.

### 2.11. Cell Viability Assay

Chicken lymphocytes were collected from the chickens on the 7th day after the third immunization. The lymphocytes were then stimulated with or without 10 μg/μL LPS for 48 h. Then, 20 μL of MTT solution was added into cells for 4 h at 37 °C, and 150 μL of DMSO was added to lyse cells. The absorbance value of OD_570_ was detected via a microplate reader, and the lymphocyte proliferation rate was calculated for each group using the following formula: lymphocyte proliferation rate (%) = (absorbance value of experimental group vs average absorbance value of control group) × 100%.

### 2.12. Ig Antibody and CD Molecular Detecion

The serum IgM and IgY antibody levels were detected one week after the three immunizations occurred, and the operation was performed according to the method provided in the ELISA kit. Additionally, the CD molecular expressions, including CD3, CD4, CD8, CD86, CD80, and CD40, were detected via qPCR, and the specific primers of these genes are shown in Table 1.

### 2.13. Statistical Analysis

Data were analyzed via one-way ANOVA using GraphPad Prism 6.01 statistical software, and Duncan’s post hoc test method was used for multiple comparisons. The results were expressed as the mean ± standard deviation error. * *p* < 0.05; ** *p* < 0.01; *** *p* < 0.001.

## 3. Results

### 3.1. MDA5-2 Protein with Complete CARD2 Domain Inhibits H9N2 AIV Replication In Vitro

To investigate the function of the MDA5 protein with CARD2 domain on the viral replication, the truncated MDA5-1 and MDA5-2 genes with different CARD2 regions were cloned from DF-1 cells, and the sizes of the clones were verified to be 534 bp and 672 bp (Figure 1C), respectively, which were consistent with the expected MDA5-1 and MDA5-2 genes. The MDA5-1 and MDA5-2 genes were constructed into the eukaryotic expression plasmids Flag-MDA5-1 and Flag-MDA5-2, which were identified via double enzyme digestion (Figure 1D) and successfully expressed after transfection into DF-1 cells, as confirmed via Western blotting with the special antibody to the Flag label in the eukaryotic vector (Figure 1E), and densitometry analysis of each band is shown in Supplementary Figure S1.

Moreover, it was observed that compared to the control with Flag-N vector transfection, the expressions of IFN-β, PKR, and MAVS in DF-1 cells transfected with Flag-MDA5-2 significantly increased (Figure 1F). Although the expression of IFN-β was slightly higher than that of the Flag-N vector control, there was no significant increased expression of PKR and MAVS in DF-1 cells transfected with the Flag-MDA5-1 plasmid (Figure 1F).

Furthermore, the expressions of viral HA, NS, and NA genes in the DF-1 cells transfected with Flag-N, Flag-MDA5-1, and Flag-MDA5-2 and inoculated with 0.1 MOI H9N2 were detected via qPCR. It was observed that the expressions of the viral HA, NS, and NA genes in DF-1 cells transfected with Flag-MDA5-2 were significantly higher than that of the Flag-N vector control (Figure 1G). However, the expressions of the viral HA, NS, and NA genes in the transfected Flag-MDA5-1 plasmid group were not significantly different to those in the control group. The above experimental results indicated that Flag-MDA5-2 with complete CARD2 domain might have a strong inhibitory effect on H9N2 AIV replication.

### 3.2. Knockout of MDA5 Gene Upregulated IFN-β Expression and Downregulated Viral M Gene, and Complementing Flag-MDA5-2 Plasmid Inhibited Replication of H9N2 Virus

**Figure 2:**
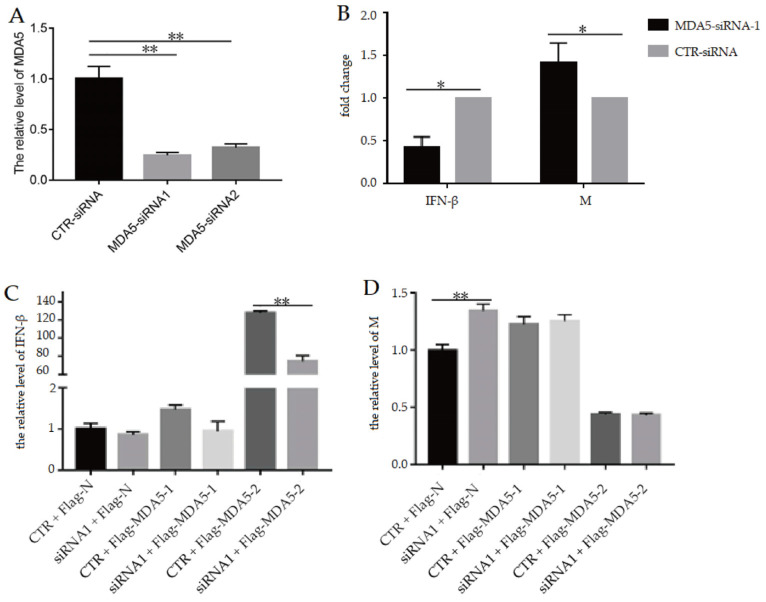
After interfering with MDA5, the complementing plasmid inhibits the replication of the H9N2 virus. (**A**) The interference effect of 100 μmol/L MDA5 interference primer, in which the ideal interference effect of MDA5-siRNA1 is shown. (**B**) The transcription level of IFN-β and the viral M gene after interfering with MDA5-siRNA1 (**C**) The transcription level of IFN-β in DF-1 cells after MDA5 gene knockout. (**D**) The level of viral M gene in DF-1 cells after MDA5 gene knockout. * *p* < 0.05; ** *p* < 0.01.

To confirm the inhibitory effect of MDA5 with CARD2 domain on the viral replication, in this paper, the interference primers for the MDA5-1 and MDA5-2 genes were designed. It was observed that the MDA5-siRNA1 and MDA5-siRNA2 primers at 100 μmol/L could effectively inhibit the expressions of MDA5-1 and MDA5-2, respectively (Figure 2A). Upon the transfection of the DF-1 cells with the MDA5-siRNA1 interfering plasmid, it was observed that the transcript levels of IFN-β were significantly upregulated compared to the control siRNA (CTR-siRNA), while the viral M gene expression was significantly downregulated (Figure 2B).

To further investigate the antiviral function of MDA5-2, DF-1 cells were transfected with the interfering plasmid to knockout the MDA5 gene and then supplemented with the Flag-MDA5-1, Flag-MDA5-2 plasmid, and Flag-N vector to detect the IFN-β expression and viral M gene after being inoculated with 0.1 MOI H9N2 AIV. It was observed that compared to the siMDA5-N (siRNA1 + Flag-N), the expression of IFN-β in DF-1 cells transfected with siMDA5-2 (siRNA1 + Flag-MDA5-2) and replenished with Flag-MDA5-2 plasmid significantly increased (Figure 2C), which was lower than that of only Flag-MDA5-2 plasmid transfection. The expression of IFN-β in DF-1 cells with siMDA5-1 (siRNA1 + Flag-MDA5-1) transfection and Flag-MDA5-1 plasmid complementation was similar to that of siMDA5-N control, with no significant difference (Figure 2C).

Furthermore, it was found that the viral M gene expressions in DF-1 cells with siMDA5-N, Flag-MDA5-1, and siMDA5-1 were increased compared to that of the Flag-N control, while M gene levels in DF-1 cells with Flag-MDA5-2 plasmid and siMDA5-2 were lower than that of the Flag-N control (Figure 2D). These results suggested that Flag-MDA5-2 might have a stronger inhibitory effect on antiviral replication than Flag-MDA5-1.

### 3.3. The Prokaryotic Expression and Purification of MDA5-1 and MDA5-2 Proteins

**Figure 3:**
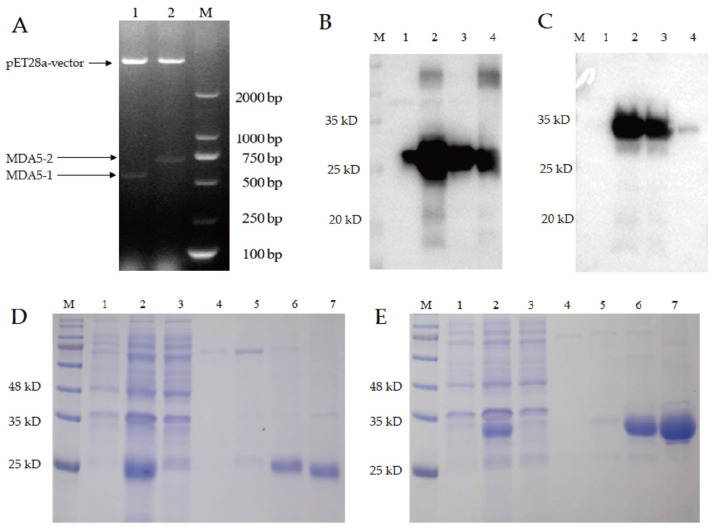
Construction of prokaryotic expression vector and expression identification of MDA5-1 and MDA5-2 proteins. (**A**) Identification of recombinant plasmids pET28a-His-MDA5-1 and pET28a-His-MDA5-2 via enzyme double digestion. (**B**) Identification of recombinant MDA5-1 protein via Western blotting. (**C**) Identification of recombinant MDA5-2 protein via Western blotting: 1—bacteria with recombinant plasmid before induction; 2—bacteria with recombinant plasmid after induction; 3—supernatant after ultrasound; 4—precipitation after ultrasound. (**D**) Purification of MDA5-1 protein. (**E**) Purification of MDA5-2 protein: 1—bacteria with recombinant plasmid before induction; 2—bacteria with recombinant plasmid after induction; 3—supernatant after filtration; 4–5—washing protein; 6–7—elution protein with 400 and 200 mM imidazole.

To investigate the immune-inducing function of the CARD2 domain of the MDA5 protein, the MDA5-1 and MDA5-2 genes were built into the prokaryotic expression vectors pET28a-His-MDA5-1 and pET28a-His-MDA5-2 (Figure 3A), which were identified via double enzyme digestion. The recombinant MDA5-1 and MDA5-2 expressions with His tag were induced via IPTG (Figure 3B,C), and we observed that the recombinant MDA5-1 protein was expressed as soluble proteins in both the supernatant and inclusion body (Figure 3B), and MDA5-2 protein was mainly expressed as soluble proteins in the supernatant (Figure 3C), with the densitometry analysis of each band shown in Supplementary Figures S2 and S3.

After being purified via His nickel column chromatography and analyzed via SDS-PAGE, it was found that both MDA5-1 and MDA5-2 proteins were adsorbed onto the nickel column, with no target protein found in the filtered supernatant. The protein purities of the recombinant MDA5-1 and MDA5-2 protein were as good when eluted at 400 and 200 mM imidazole with fewer heteroproteins (Figure 3D,E).

Furthermore, it was found that there was no target protein in the supernatant after filtration, indicating that it might have been well bound to the nickel filler, and the high concentration eluent could elute the target protein with less impurity protein.

### 3.4. MDA5 Protein with Complete CARD2 Domain Induced Strong Antibody Production in the Immunized Chickens

**Figure 4:**
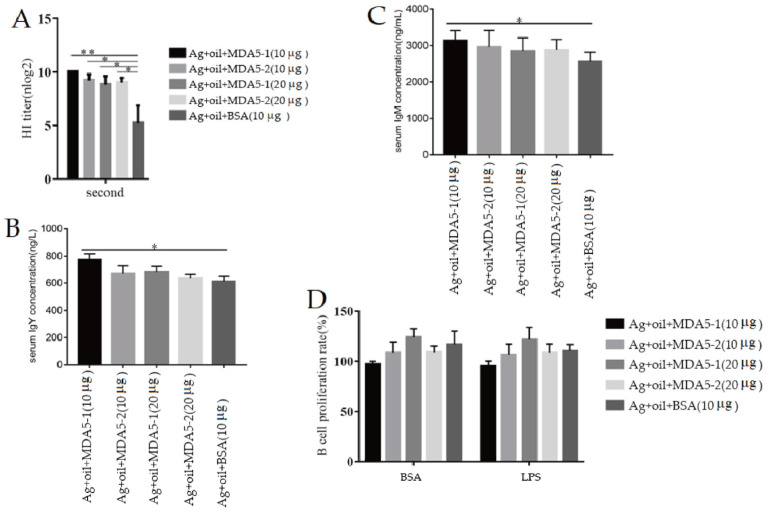
Antibody levels in the recombinant protein immunization group. (**A**) HI antibody level. The inactivated vaccines containing the purified recombinant were thoroughly mixed with white oil adjuvant. Chickens were immunized with the prepared vaccines mixed with H9N2 antigen plus recombinant MDA5-1 and MDA5-2 proteins and white oil adjuvant, and HI antibody titer was detected on the 14th day after the second immunization. (**B**) IgY level in chicken serum after the third immunization. (**C**) IgM level in chicken serum after the third immunization. (**D**) The proliferative activity of chicken lymphocytes. * *p* < 0.05; ** *p* < 0.01.

To detect the effect of MDA5 protein with CARD2 domain on the humoral immune response in vivo, experimental vaccines containing the truncated MDA5-1 and MDA5-2 proteins were subcutaneously injected into chickens. It was found that on the 14th day after the second immunization, the HI antibody titers of chickens immunized with vaccine plus 10 μg and 20 μg recombinant MDA5-1 proteins were significantly increased compared to the antigen control. Additionally, the immune groups with MAD5-2 protein at 10 μg and 20 μg produced significantly increased HI antibody levels compared to that of the antigen control (Figure 4A).

Also, on the 7th day after the third immunization, chickens immunized with 10 μg recombinant MDA5-1 and MDA5-2 proteins produced significantly higher levels of IgY antibodies compared to the antigen control (Figure 4B). However, IgY levels in 20 μg recombinant MDA5-2 protein immunization groups were higher than that of the antigen control, with no significant difference.

Furthermore, compared to the antigen control, the IgM levels derived from chickens immunized with recombinant MDA5-1 MDA5-2 proteins at 10 μg and 20 μg were increased (Figure 4C), in which the IgM antibody level in 10 μg MDA5-1 protein immunization group was highest among all of the immunization groups. These results suggested that MDA5 protein with partial and complete CARD2 domains might present strong potential to induce the secretion of antibodies during vaccination.

To assess the viability of lymphocytes, chicken lymphocytes were stimulated with LPS and BSA for 48 h and detected using the MTT method. As shown in Figure 4D, in both BSA treatment groups, except that the lymphocyte viability of antigen plus 10 µg MDA5-1 was lower than that of the antigen control, the lymphocyte viabilities of the three other groups did not significantly differ compared to that of the antigen control. Furthermore, the tendency of lymphocytes viabilities in LPS treatment was similar to that of BSA treatment in all immune groups. Also, it was found that the lymphocyte viability was lowest in the group immunized with 10 μg MDA5-1 protein and highest in the group immunized with 20 μg MDA5-1 protein among all of the groups. These results suggest that the recombinant MDA5-1 and MDA5-2 immunization might not have cytotoxic effects on chicken lymphocytes.

### 3.5. MDA5 Protein with CARD2 Domain Induced the Increased Expression of Surface Molecules on Lymphocytes during Immunization

**Figure 5:**
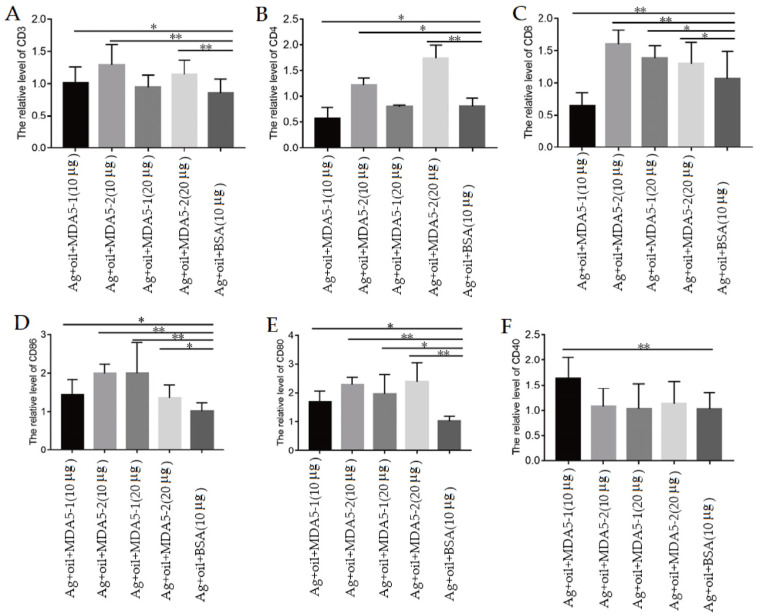
MDA5 protein induced the increased expression of surface molecules on lymphocytes during immunization. (**A**) The expression level of CD3. (**B**) The expression level of CD4. (**C**) The expression level of CD8. (**D**) The expression level of CD86. (**E**) The expression level of CD80. (**F**) The expression level of CD40. * *p* < 0.05; ** *p* < 0.01.

In order to further verify the immunoregulatory ability of MDA5 protein within the CARD2 domain, the mRNA levels of the CD3, CD4, and CD8 molecules in the spleen lymphocytes of the immunized chickens were detected via qPCR on the 7th day after three immunizations. It was observed that compared to that of the antigen control, the CD3 and CD4 expressions levels in chickens immunized with MDA5-2 proteins were significantly increased (Figure 5A,B). Additionally, except for 10 μg MDA5-1 protein immunization group, chickens immunized with the vaccine plus MDA5-1 and MDA5-2 proteins all produced higher levels of CD8 expression than that of the antigen control (Figure 5C).

Furthermore, it was found that CD86 levels from chickens immunized with the vaccine plus MDA5-1 and MDA5-2 at 10 μg and 20 μg were higher than that of the antigen control (Figure 5D), in which the CD86 levels in the 10 μg MDA5-2 and 20 μg MDA5-1 groups were highest. Additionally, compared to that of the antigen immunization control, chickens immunized with MDA5-1 and MDA5-2 at 10 μg and 20 μg produced significantly increased CD80 expressions (Figure 5E), in which the expression levels of CD80 in MDA5-2 groups at 10 μg and 20 μg were higher than those of MDA5-1 groups. Unexpectedly, only the CD40 expression level in chickens immunized with 10 μg MDA5-1 was significantly higher than that of the antigen control (Figure 5F). These results implied that MDA5 with partial and complete CARD2 domains could induce the surface molecule expression of lymphocytes during immunization. The functional mechanism of the CARD2 domain in MDA5 proteins may need further exploration.

## 4. Discussion

MDA5 is an important pattern recognition receptor, which plays a crucial role in identifying the pathogenic micro-organisms and initiating cellular innate immunity [24]. The overexpression of the CARD region of duck MDA5 can activate the IRF-7 signaling pathway, induce the activation of the IFN-β promoter through a cascade reaction, and upregulate the mRNA expression of antiviral molecules, such as PKR and Mx, as well as pro-inflammatory cytokines such as IL-6 and IL-2 [25].

Chicken MDA5 is a critical receptor that mediates the antiviral response triggered by short dsRNA and AIV in the absence of RIG-I in chickens [10], thereby playing a vital role in resisting viral infections. Therefore, the research objectives of this study were to investigate the antiviral and immune enhancement properties of truncated MDA5 genes with different CARD2 regions during H9N2 avian influenza virus infection and vaccination. In this paper, MDA5-1 with partial CARD2 regions and MDA5-2 with complete CARD2 regions were designed based on the genetic constitution of chicken MDA5. It was found that compared to that of MDA5-1, the over-expression of MDA5-2 not only significantly stimulated the upregulated expression of the antiviral molecule IFN-β and PKR, but also strongly inhibited the expression of viral proteins HA, NA, and NS. The CARD region interacts with the CARD region of the downstream MAVS to form a complex, which activates the downstream pathway to induce the expression of the IFN-I gene and antiviral molecules [26,27]. The overexpression of chicken MAVS can induce the production of type I IFN [28]. Also, it was found that the overexpression of MAD5-2 enhanced the expression of MAVS. These results suggest that the integrity of CARD2 domain is crucial for the antiviral function of MAD5.

To further investigate the association between the CARD2 domain and antiviral function of MDA5, in this paper, RNA interference technology was used to knockdown MDA5 genes in DF-1 cells. The results showed that the expression of IFN-β decreased after the knockdown of MDA5 genes, and the replenishment of Flag-MDA5-2 could upregulate the expression of IFN-β. These results indicated that chicken MDA5 might be involved in the process of recognizing AIV and activating immune response, and the integrity of CARD2 region might be crucial for the IFN-β pathway process. However, there was no significant difference in virus replication between the Flag-MDA5-2 group and the control group after the knockdown of MDA5. There were the following explanations: When the gene is not knocked down, the whole pathway is intact, and IFN-β induced by the backfill plasmid and infection with H9N2 is definitely higher than that of the knockdown group, which is strictly controlled by the cells. Chicken cells lack RIG-I gene, and MDA5 is a PRR that recognizes viral RNA. After the knockdown of MDA5, virus recognition and downstream signal transduction might be inhibited, even if IFN-β induced by the backfill plasmid is still limited. These results suggest that CARD2 region has the ability to inhibit the replication of the H9N2 virus. However, the mechanism of the CARD2 domain involved in antiviral function of MDA5 on virus replication needs to be further explored in future experiments.

Furthermore, the adjuvant properties of MDA5 have barely been reported. To investigate the immune-inducing function of the CARD2 domain derived from MDA5, in this paper, the chickens were immunized with MDA5-1 and MDA5-2, accompanied by an avian influenza inactivated antigen. It was observed that the expression levels of CD80 and CD86 mRNA were higher in the MAD5-1 and MDA5-2 protein combined immunization group than that of the antigen control group. CD80 and CD86 are co-stimulatory molecules primarily expressed on the surface of antigen-presenting cells and bind to CD28 or CD152 after antigen stimulation to promote the activation of T cells [29]. The lack of CD80 and CD86 can lead to the inability to activate T cells, which will affect the antigen-presenting ability of APC and the body’s immune system [30]. These results suggest that the recombinant proteins MAD5-1 and MAD5-2 can stimulate antigen immunization to produce effective CD86 and CD80 expression, indicating that they can effectively stimulate antigen presentation in the process of antigen immunization. However, the mechanisms of CARD2 domain in MDA5 on antigen presentation need to be further explored.

It is well known that CD3, CD4, and CD8 are mainly expressed on the surface of T cells and are CD molecules involved in recognition, adhesion, and activation [31]. CD3 forms a complex with T cell surface receptors and transmits signals into cells to initiate T cell activation [32]. CD4 molecules help to identify antigens presented by MHC class II molecules, and CD8 molecules assist in the identification of antigens presented by MHC class I molecules [33]. In this paper, the recombinant MAD5-1 and MAD5-2 proteins could induce the mRNA levels of CD3, CD4, and CD8 during antigen immunization, and the effect of MAD5-2 on stimulating T cell response was stronger than that of MAD5-1. 

CD154 is mainly expressed on the surface of activated CD4+ T cells [34]. CD154 contributes to the interaction between B lymphocytes and T lymphocytes, and it can also bind to CD40 on the surface of APC cells to promote the expression of the co-stimulatory molecules CD80/CD86 or cytokines [35]. The results of this study confirmed that MAD5-1 and MAD5-2 combined with inactivated virus immunization stimulated the expression of CD40 and CD154 during vaccination. These results suggested that MDA5 with the CARD2 domain might have the potential to induce the antigen to activate the immune process.

Based on the antiviral and immune-inducing function detailed in this study, further investigations are warranted to elucidate the specific molecular mechanisms via which MDA5-2, containing complete CARD2 region, activates the interferon pathway and inhibits the replication of the H9N2 influenza virus. Understanding the precise signaling pathways and down-stream effector molecules involved can provide insights into the antiviral functions of MDA5 and potentially identify novel targets for therapeutic intervention. Furthermore, it will be beneficial to investigate the immune enhancement roles of MDA5 in other subtypes or strains of AIV, as well as in other RNA viruses. Assessing the broader spectrum of MDA5’s antiviral and immune enhancement functions can provide insights into its potential applications in combating different viral infections.

## 5. Conclusions

MAD5 plays an important role in antiviral and immune enhancement. In this study, MDA5-2 containing a complete CARD2 region activates the interferon pathway and inhibits the replication of the H9N2 influenza virus. Also, the knockdown of MDA5 genes confirmed that MDA5-2 has a strong antiviral effect on the H9N2 virus. Furthermore, the antibody levels and expression of surface molecules on T and B cells in immunized chicken proved that MDA5-1 and MDA5-2 can act as immune enhancers to enhance the humoral immune, cellular-mediated immune, and antigen presentation response to the H9N2 antigen. These findings hold significant implications for comprehending the antiviral mechanism of MDA5 with the complete CARD2 domain and developing the novel immune adjuvants used in the AIV vaccine.

## Figures and Tables

**Table 1:** Primers used in this study.

Primers	Sequence (5′-3′)
Flag-MDA5-1	F: AAGCTTATGTCGGAGGAGTGCCGAGACGAGC (BamH I)
	R: GTCGACTTACTGAGAGAACCAGTCCTTCTTC (Sal I)
Flag-MDA5-2	F: AAGCTTATGTCGGAGGAGTGCCGAGACGAGC (BamH I)
	R: GTCGACTTATACTGGTTGGCTTGCAGCTTCT (Sal I)
His-MDA5-1	F: GGGATCCATGTCGGAGGAGTGCCGAGACGAGC (BamH I)
	R: CCAAGCTTTTACTGAGAGAACCAGTCCTTCTTC (Hind III)
His-MDA5-2	F: CGGGATCCATGTCGGAGGAGTGCCGAGACGAGC (BamH I)
	R: CCAAGCTTTTATACTGGTTGGCTTGCAGCTTCT (Hind III)
β-actin	F: GAGAAATTGTGCGTGACATCA
	R: CCTGAACCTCTCATTGCCA
β-IFN	F: CCTCCAGCTCCTTCAGAATAC
	R: GTGCGGTCAATCCAGTGTT
MAVS	F: CACCCACGAGGTCCATGTG
	R: TGCTTCATCTGGGACATCATTG
PKR	F: TATGGTACAGGCGTTGGTAAG
	R: TATGGTACAGGCGTTGGTAAG
HA	F: TTACCCTGTTCAAGACGCCC
	R: GCCACACTCGTTGTTGTGTC
NS	F: GGTGATGCCCCATTCCTTGA
	R: TTGCTTTCCCGCACGAGTAG
NA	F: GGCGACACACCAAGAAATGA
	R: AACCTGAGCGTGAATCCACTT
M	F: TCCTCTCGTTGTTGCAGCAAGTATC
	R: ATAGACTCAGGCACTCCTTCCGTAG
MDA5-siRNA1	GCUGCAAGCCAACCAGUAUTT
MDA5-siRNA2	GCAUUUACGAAAGGAGUUUT
MAVS-siRNA1	GCUGUGAGCUCGGAUGUUUTT
CTR-siRNA	AGGUAGUGUAAUCGCCUUG
ChMDA5	F: GGACGACCACGATCTCTGTGT
	R: CACCTGTCTGGTCTGCATGTTATC
ChMAVS	F: CACCCACGAGGTCCATGTG
	R: TGCTTCATCTGGGACATCATTG
CD3	F: GGACGCTCCCACCATATCAG
	R: AAGCTCGTGACATGAGTCCC
CD4	F: TGTGGAACTGTCACCTCGTG
	R: CACATGCATGCAAGGCCAAT
CD8	F: GCTGTACTTCAGCTCGGGAC
	R: ATGTCCTTGTTGACGTGGCT
CD40	F: AGGCACCTTCTCCAATGTATC
	R: GTCCCTTTCACCTTCACCAC
CD80	F: TGTGACCCTCTTTGTCACCG
	R: TGTGACCCTCTTTGTCACCG
CD154	F: TGCAGAAATGTCAGACGGGA
	R: CAACTCCTCACTGGCTGTCC
CD86	F: ACCAGCAAGCTGAATATCCCA
	R: GACTAGCGGCACTGAGACAA

## Data Availability

The data presented in this study are available upon request from the corresponding author.

## Supplementary Materials

The following supporting information can be downloaded at: https://www.mdpi.com/article/10.3390/vaccines11101542/s1, Figure S1. The eukaryotic expression identification of MDA5-1 and MDA5-2 protein; Figure S2. The expression identification of prokaryotic plasmids pET28a-His-MDA5-1; Figure S3. The expression identification of prokaryotic plasmids pET28a-His-MDA5-2.
