# Supplementary material for: MDA5 with Complete CARD2 Region Inhibits the Early Replication of H9N2 AIV and Enhances the Immune Response during Vaccination
Source: Vaccines (Basel). 2023 Sep 28;11(10):1542. doi: 10.3390/vaccines11101542 (PMC10611263; doi:10.3390/vaccines11101542)
Supplement: Supplementary file 1 [file vaccines-11-01542-s001.zip › vaccines-2567675-supplementary.pdf]

Supplemental Figure legend

Supplemental Figure 1

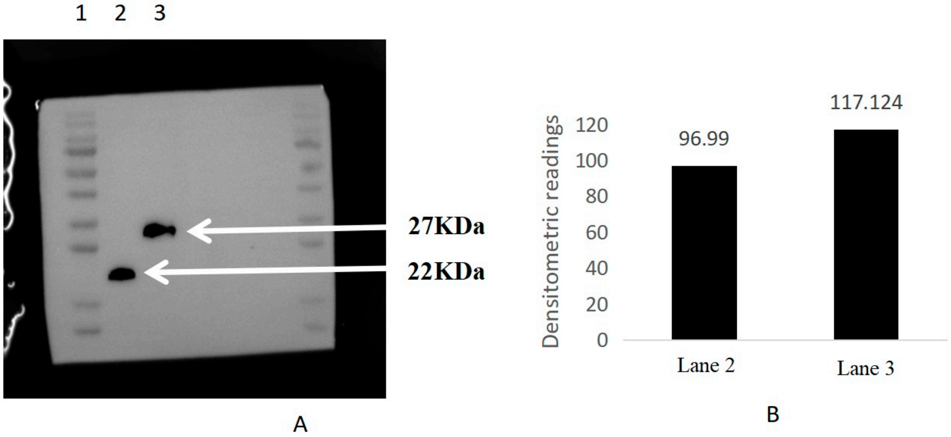

Supplemental Figure 1. The eukaryotic expression identification **of MDA5-1 and MDA5-2 protein.** (A) The whole blot (uncropped blots) showing all the bands with all molecular weight markers. Lane 1: Marker; Lane 2: The eukaryotic expression of MDA5-1 protein (22 KDa); Lane 3: The eukaryotic expression of MDA5-2 protein (27 KDa). (B) The densitometry analysis of each band in supplemental figure 1A.

Supplemental Figure 2.

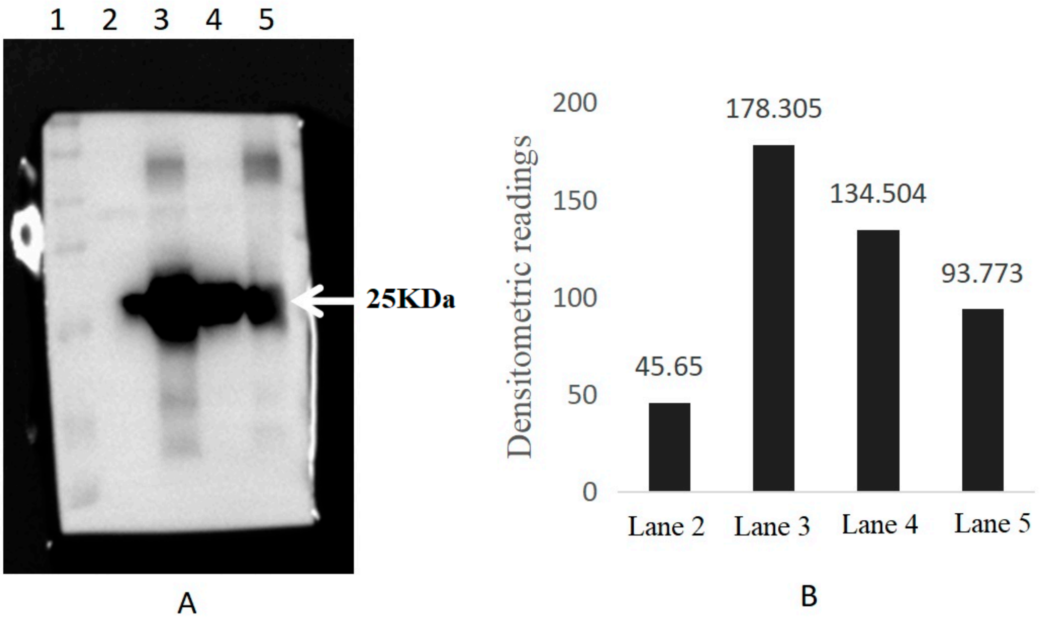

Supplemental Figure 2. The expression identification of prokaryotic plasmids pET28a-His-MDA5-1. (A) The whole blot (uncropped blots) showing all the bands with all molecular weight markers **(25 KDa)**. Lane 1: Marker; Lane 2: Bacteria with recombinant plasmid before induction; Lane 3: Bacteria with recombinant plasmid after induction; Lane 4: Supernatant after ultrasound; Lane 5: Precipitation after ultrasound. (B) The densitometry analysis of each band in supplemental figure 2A.

Supplemental Figure 3.

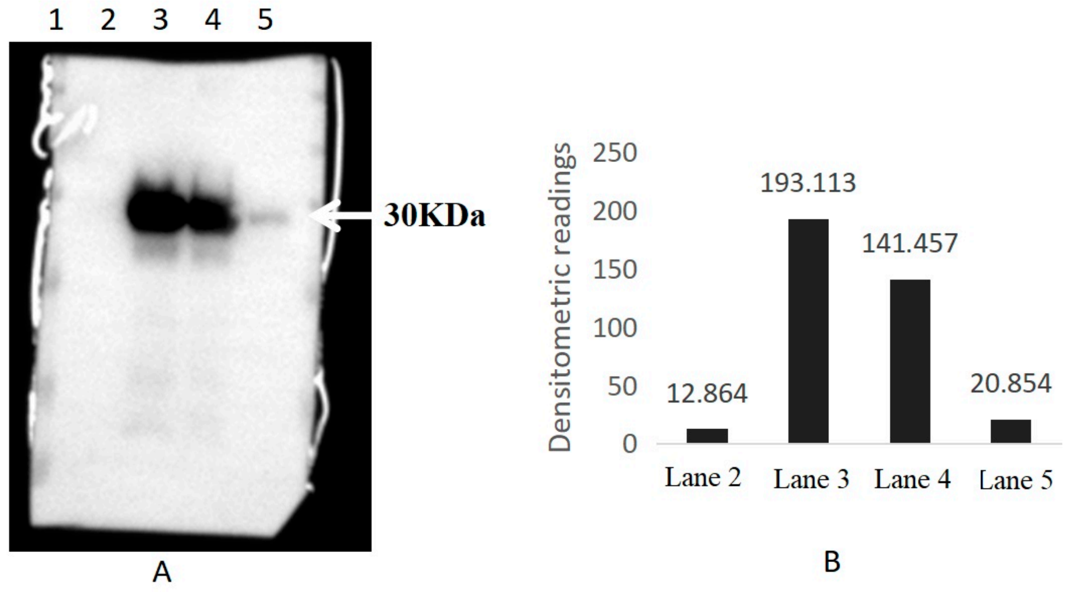

Supplemental Figure 3. The expression identification of prokaryotic plasmids pET28a-His-MDA5-2. (A) The whole blot (uncropped blots) showing all the bands with all molecular weight markers (30 KDa). Lane 1: Marker; Lane 2: Bacteria with recombinant plasmid before induction; Lane 3: Bacteria with recombinant plasmid after induction; Lane 4: Supernatant after ultrasound; Lane 5: Precipitation after ultrasound. (B) The densitometry analysis of each band in supplemental figure 3A.
